# A woman with schizophrenia who died due to Takotsubo cardiomyopathy occurring after electroconvulsive therapy

**DOI:** 10.1186/s12888-024-05593-7

**Published:** 2024-02-19

**Authors:** Takahiro Muto, Hotsumi Kyono

**Affiliations:** Yokohama City Minato Red Cross Hospital, Yokohama, Japan

**Keywords:** Case report, Electroconvulsive therapy, Schizophrenia, Takotsubo cardiomyopathy

## Abstract

**Background:**

Electroconvulsive therapy (ECT) is a safe treatment for treatment-resistant schizophrenia. However, it has some side effects, and Takotsubo cardiomyopathy is considered one of the minor complications. Several cases of patients developing Takotsubo cardiomyopathy during a course of ECT have been reported, but none have died. We present a case of post-ECT Takotsubo cardiomyopathy that became fatal.

**Case presentation:**

We experienced a case of a 67-year-old woman who had delusions and catatonic symptoms due to schizophrenia but was resistant to several medications. Her symptoms improved by conducting ECT, but she had difficulty maintaining her improvement, which caused her to receive multiple courses of ECT. 3 weeks after her 6th course of ECT, the patient was diagnosed with Takotsubo cardiomyopathy and had a fatal outcome.

**Conclusion:**

Our patient had numerous cases of aspiration pneumonia and malnutrition before ECT was performed, which might have made this case fatal. In conclusion, appropriate supplementation of nutrition and reduction of physical stressors are important to avoid death from Takotsubo cardiomyopathy caused by ECT. Prescribing clozapine was a solution in the present case, but there are some difficulties, such as the restriction against prescribing this drug in Japan.

## Background

The outcome of schizophrenia patients has several patterns. One study [1] reported that in a 25 -year follow-up, approximately 50% of surviving cases had favorable outcomes, but another study [2] claimed that 23% of the patients became treatment resistant after a 10-year follow-up.

There are several ways to manage treatment-resistant schizophrenia, and one is electroconvulsive therapy (ECT). When ECT and antipsychotic medication are combined, compared with antipsychotic medication alone, the standard mean difference in symptomatic improvement was found to be -0.67, and the risk ratio of remission rate was 2.2, with a number needed to treat of 8 [3]. This result proves the effectiveness of ECT. ECT is also well known as a safe treatment [4], but there are some side effects. Major side effects include headaches and memory impairments [3]. There are also other side effects, and Takotsubo cardiomyopathy is thought to be one of the minor complications. According to a study [5] that analyzed case reports of patients developing Takotsubo cardiomyopathy when ECT was conducted, all patients recovered from Takotsubo cardiomyopathy, which means that none of the patients who developed Takotsubo cardiomyopathy after ECT was completed or died after developing Takotsubo cardiomyopathy.

Here, we present a patient who died from Takotsubo cardiomyopathy that occurred after multiple courses of ECT.

### Case presentation

This case involved a Japanese woman (Mrs. A) who graduated from the vocational school of sewing but was never employed and lived with her parents. When she was 35 years old, she started to have auditory hallucinations and insomnia. Medication was started, but 5 years later, she cried out that she was being targeted by someone and performed strange actions such as watering her house for no reason or going out with bare feet. She was diagnosed with schizophrenia at B Hospital. After 3 years of hospitalization, she became an outpatient of B Hospital, but she quit her medication. She thereafter became irritated and rejected any necessary treatment: consequently, she was hospitalized at B Hospital when she was 53 years old. She was prescribed a maximum dose of 24 mg haloperidol, 200 mg chlorpromazine, 9 mg bromperidol, 9 mg risperidone, 48 mg perospirone, 10 mg olanzapine, and 24 mg aripiprazole, but none of these drugs were effective.

The patient first came to our hospital when she was 56 years old. At that time, she was screaming that she heard a baby crying and believed that our hospital was on fire. She also had catatonic symptoms such as akinesia and making weird postures. Her weight was 45.5 kg, and her body mass index (BMI) was 18.9 kg/m^2^. She had a comorbidity of diabetes mellitus. She had her first course of ECT, and after 10 treatments, she became calm. After the ECT was over, she returned to B Hospital for further treatment.

Subsequently, she occasionally became catatonic such as lying beside the toilet and stay still with her hands up or staring the ceiling, and refused to eat or take medication; thus, she returned to our hospital several times, having 5 courses of ECT in total. Despite this treatment, her delusions and negative symptoms worsened gradually. Electrocardiogram was examined several times at our hospital and there were no abnormal findings except for sinus tachycardia.

At the age of 64, Mrs. A repeatedly developed aspiration pneumonia due to nasogastric tube insertion when she refused to eat. She began losing weight, and by the time she became 67 years old and came to our hospital for the 6th time in April, she weighed only 27.8 kg, and her BMI was 12.6 kg/m^2^. She was silent and nodded to any question she was asked when she was admitted, but after hospitalization, she started screaming and refused to eat, so we started injecting 10 mg of haloperidol intravenously every day. Since she had the complication of aspiration pneumonia, we also treated this disease by intravenous injection of antibiotics. Her aspiration pneumonia improved, but even after her intravenous injection of haloperidol was increased to 20 mg, she exhibited hostile attitudes to us, such as yelling “your breath stinks!”, shouting “be quiet!” or being catatonic, such as holding a toothbrush in her mouth for about 30 min. We performed a 6th course of ECT to manage her resistance to treatment and improve her catatonia. We conducted 3 bilateral ECT treatments a week. Intravenous injection of 20 mg or 25 mg of propofol and 20 mg of suxamethonium was used for anesthesia. When her blood pressure was elevated after ECT, 1 to 2 mg of nicardipine was injected intravenously. The mean duration of convulsion was 48 s. The mean heart rate and blood pressure before ECT and during convulsion were 93 bpm and 149/65 mm Hg, 123 bpm and 154/82 mm Hg, respectively. A total of 14 ECT treatments and a prescription of 20 mg asenapine made her able to eat again, although she occasionally thought that there was something in her meals that weakened her muscles. She was able to stay calm and did not refuse to take her medication. She finished her fourteenth treatment of ECT in May 24 and returned to hospital B in May 27. There were no abnormalities in her electrocardiograms during hospitalization in our hospital, and she did not complain of symptoms suggesting Takotsubo cardiomyopathy, such as chest pain.

Mrs. A started to refuse eating in June, and on June 13, she had several hypoglycemic attacks, and her oxygen saturation decreased to 77% in room air. She came to our hospital on June 14 to receive physical treatment as well as treatment for schizophrenia.

**Fig. 1:**
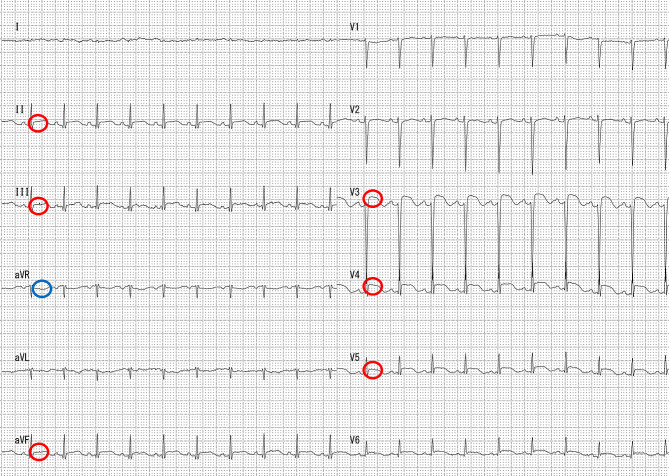
Electrocardiogram of the case. The red circle shows the ST elevations and the blue circle shows the ST depression

**Fig. 2:**
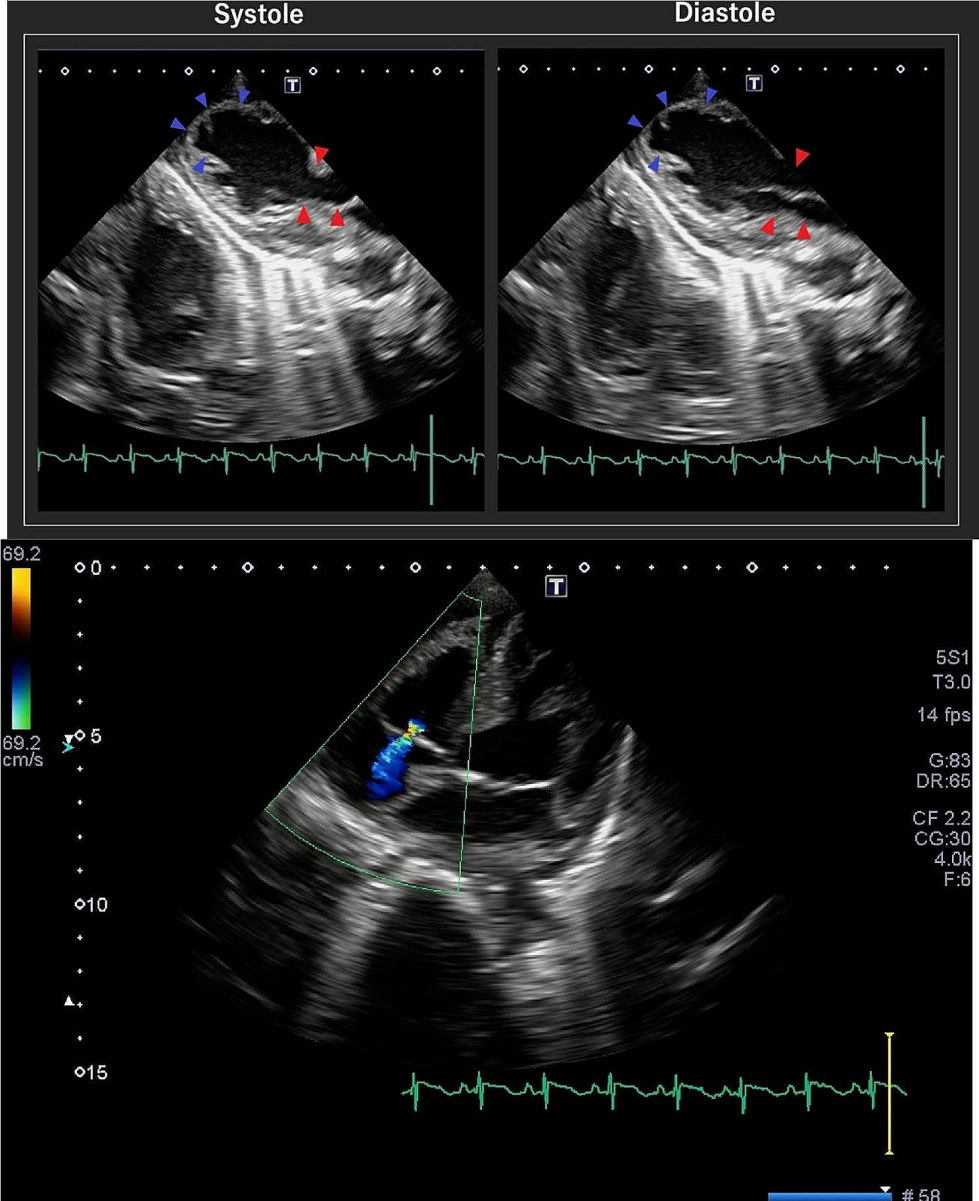
Echocardiogram of the case. The red arrow shows the hyperkinesis of the basal walls and the blue arrow shows the akinesis of the apex

**Fig. 3:**
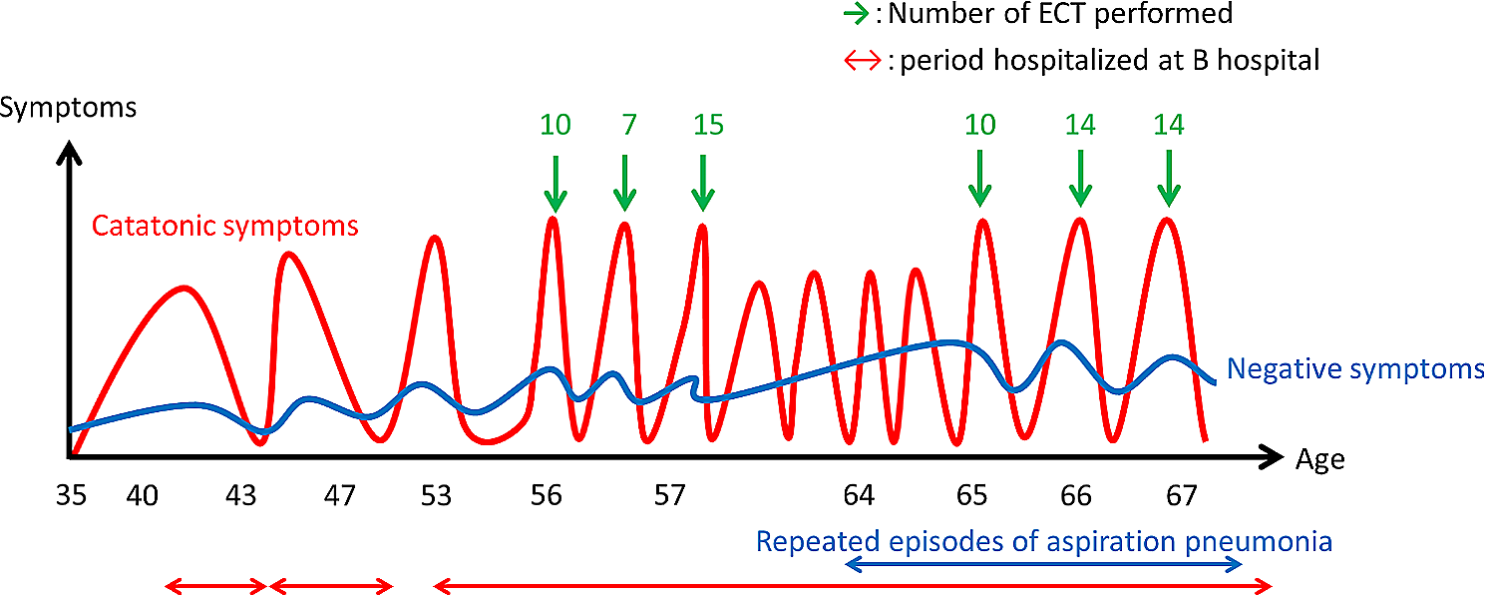
The clinical progress of Mrs. A

**Fig. 4:**
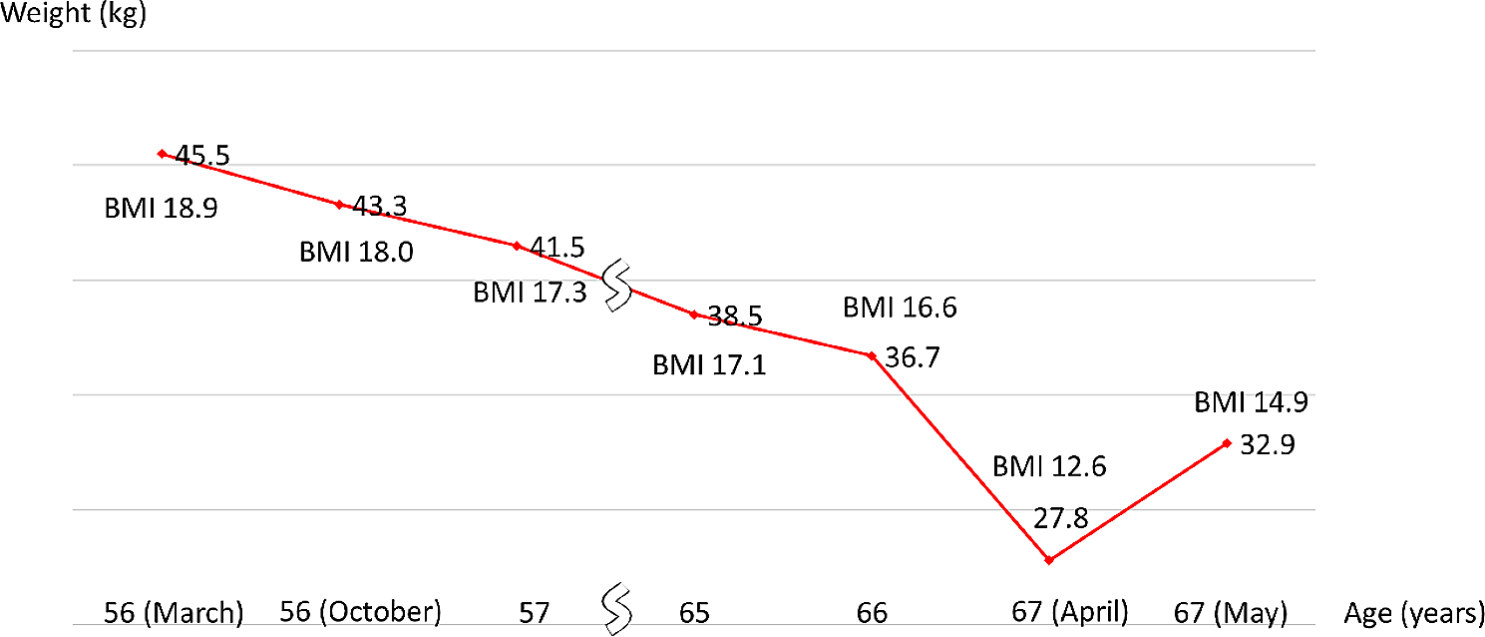
The change in weight in Mrs. A

We checked her electrocardiogram (Fig. 1) and found several ST elevations, so we conducted an echocardiogram (Fig. 2). Since there was akinesis of the apex, hyperkinesis of the basal walls leading to low output, we diagnosed the patient with Takotsubo cardiomyopathy. We denied that she developed aspiration pneumonia because there were no findings in her blood test and chest X-ray that suggests development. She had numerous hypoglycemic attacks and pressure drops on June 15, so she was prescribed a vasopressor drug. She once became able to talk but her chest X-ray showed that she was developing pulmonary congestion. She started to lose consciousness on June 21 and passed away on June 22 due to CO_2_ narcosis. Pathologic autopsy was not conducted since her family disagreed. Figures 3 and 4 show her clinical course and her change in weight, respectively.

## Discussion

Takotsubo cardiomyopathy is a syndrome characterized by acute transient left ventricular systolic dysfunction, which can be challenging to distinguish from acute myocardial infarction but does not involve obstructive coronary arteries or plaque rupture [6]. Common symptoms of Takotsubo cardiomyopathy are chest pain and dyspnea, but some patients are diagnosed by chance when new changes are found in an electrocardiogram [7]. Most of the patients are elderly women, and this syndrome is thought to be caused by physical or emotional stress [6]. The most famous theory of Takotsubo cardiomyopathy development is myocardial injury caused by enhanced levels of catecholamines [6]. The mortality rate during hospitalization is 2.6% [8], and 5.6% of patients die from any cause [6]. Most deaths occur within a year [9] and are not from cardiac causes [10]. The predictors of death within a year include physical stress, a history of depression or cancer, and increased age [10]. The highest mortality rate is seen in patients with Takotsubo cardiomyopathy caused by physical stress, and the lowest mortality rate is seen in patients with Takotsubo cardiomyopathy caused by mental stress [11].

According to a study that analyzed 24 patients who developed Takotsubo cardiomyopathy during ECT [5], 21 patients were women, and the mean age was 62.3 years. Sixteen patients had major depressive disorder, 5 patients had bipolar disorder, and 3 patients had schizophrenia. Our case involved a woman who was close to the mean age of developing Takotsubo cardiomyopathy. According to case reports [5], 17 patients developed Takotsubo cardiomyopathy by the 3rd course of ECT. Although we were not able to conduct a ventriculography or coronary angiography, we still think that we were able to make differentiation from acute myocardial infraction for our patient from her electrocardiogram. One study [12] shows that a combination of having a ST depression in lead aVR and lacking of ST elevation in lead V1 will identify Takotsubo cardiomyopathy with 91% sensitivity, 96% specificity, and 95% predictive accuracy, and our patient’s electrocardiogram has both findings. Our patient developed Takotsubo cardiomyopathy after the 6th course of ECT, but because the 4th course was performed 8 years after the 3rd course, the period of Takotsubo cardiomyopathy development in our case was close to that in the majority of the cases reported, excluding the fact that the development occurred after the end of the course of ECT. Although 10 patients were reported to develop cardiogenic shock or abnormal vital signs related to cardiogenic shock [5], none of the patients died after developing Takotsubo cardiomyopathy. These results show that we might be the first to report a fatal case of post-ECT Takotsubo cardiomyopathy.

Our patient developed aspiration pneumonia repeatedly 3 years before she died. She weighed 36.7 kg 4 months prior to the 6th visit to our hospital, meaning that she lost 6.9 kg within 4 months, as shown in Fig. 4. She also had several hypoglycemic attacks before she came to our hospital for the final time. In addition to these physical stresses, she underwent ECT, which is said to release adrenaline and dopamine [13], making her more likely to develop Takotsubo cardiomyopathy. Since seizures are reported as risk factors of Takotsubo cardiomyopathy [7], and considering the fact that ECT is a treatment that causes numerous convulsions frequently, we think that ECT might have been the cause of Takotsubo cardiomyopathy.

According to a report [14] that analyzed 48 cases of patients who developed Takotsubo cardiomyopathy after epilepsy, 25 patients were diagnosed with Takotsubo cardiomyopathy more than 5 h after the seizure, the mean and the longest time interval being 50 and 288 h, respectively. Another study [15] that observed the relationship between acute psychological stress and incidence of Takotsubo cardiomyopathy by observing 8 hospitals of Nigata for 4 weeks after a strong earthquake which struct that region on 2004, the number of patients increased significantly until the third week compared with the corresponding periods in 2002 and 2003. These results show that Takotsubo cardiomyopathy caused by ECT might develop several days later and suggest the possibility that stress which triggers Takotsubo cardiomyopathy might take approximately 3 weeks to actually develop Takotsubo cardiomyopathy. Since our patient conducted her final treatment of ECT on May 24 and she was diagnosed with Takotsubo cardiomyopathy on June 14, which is 3 weeks after the final treatment of ECT, we think that stress due to ECT could trigger Takotsubo cardiomyopathy in our case. We are thinking of another possibility that she developed Takotsubo cardiomyopathy earlier than June 14 and the drop of oxygen saturation in June 13 was caused by a complication of Takotsubo cardiomyopathy since some patients develop Takotsubo cardiomyopathy lacking clinical symptoms [7]. But this hypothesis cannot be concluded because no tests that suggest the possibility of developing Takotsubo cardiomyopathy was performed after she went to B Hospital and this is the limitation of our report. We think her CO_2_ narcosis was caused by pulmonary congestion due to low ejection fraction which is a symptom of Takotsubo cardiomyopathy.

Our patient’s symptoms did not improve with several antipsychotics and although ECT was effective to catatonic symptoms, which was the reason we performed several courses, the duration of remission was insufficient, so using clozapine before ending up conducting ECT several times was a better way of treating her symptoms. Clozapine is prescribed when 2 or more antipsychotics are not effective, and 70% of patients have improved symptoms when clozapine is used [16]. However, clozapine has several life-threatening side effects, such as neutropenia, myocarditis, and gastrointestinal hypomotility [16], so by the time she became 67, Mrs. A’s death might not have been avoided even by starting clozapine. Instead, she should have been considered for treatment with clozapine in her younger years, which would have made her usage of clozapine much safer. The reason our patient was not able to benefit from clozapine might be the low prescription rate of clozapine in Japan, which is said to be prescribed to only 0.2% of schizophrenia patients receiving antipsychotics [17]. This low rate might occur due to the rules for prescribing clozapine in Japan, which restrict clozapine prescriptions to only approved institutes that are registered as part of the Clozaril Patient Monitoring Service (CMPS) [18]. Since B Hospital and our hospital were not registered as a CMPS, none of the doctors who treated her were able to prescribe clozapine even though he or she wanted to and this is the cause she had to take multiple courses of ECT even though the treatment was insufficient.

## Conclusion

Not only did her physical stresses before diagnosis of Takotsubo cardiomyopathy make our patient susceptible to the highest possibility of dying within a year, but she also had several physical stressors after diagnosis, such as hypoglycemic attacks, pressure drops, and CO_2_ narcosis. Because most patients with Takotsubo cardiomyopathy die from noncardiac causes, her death might have been unavoidable by the time she was diagnosed.

In sum, Mrs. A’s death might have been avoided by reducing her physical stress, such as improving her nutritional status or starting clozapine before conducting several courses of ECT to manage her resistance to treatment due to schizophrenia.

## Data Availability

All data necessary are included in this article.

## Abbreviations

BMI: Body Mass Index
CMPS: Clozaril Patient Monitoring Service
ECT: Electroconvulsive therapy
